# Surgery

**Published:** 1893-03

**Authors:** 


### SURGERY.

EDITED BY DR. FLOYD W. McRAE.

### TREATMENT OF ISCHIO-RECTAL ABSCESS.

In this form of abscess the purulent matter occupies the loose cellular tissue of the ischio-rectal fossa in close relation to the rectum, and from the anatomical peculiarities of the tissue in which it is situated it is apt to burrow widely and, if left to itself, to open into the rectum or through the skin into the region of the anus, and result in the production of one or other forms of fistula-in-ano, either the complete form or the external or internal incomplete form of this affection.

To obviate this unfortunate result the prompt treatment of ischio-rectal abscess is urgently demanded, and I am decidedly of the opinion that attempts at abortive treatment of this form of abscess are worse than useless; that by such treatment valuable time is lost, and the surgeon has finally to resort to surgical treatment after extensive burrowing of pus has occurred with possibly perforation of the wall of the rectum.

It is, and has been for a long time, a surgical axiom that an ischio-rectal abscess should be opened promptly, and if so treated the probability of a fistula-in-ano resulting is much diminished. I formerly was satisfied to open these abscesses by a small incision, evacuate the pus, and in many cases a prompt recovery took place without the formation of a fistula, but in others a fistula resulted; whether the rectal communication was present at the time of opening or resulted from the imperfect drainage secured by a small incision I am unable to say, but I am sure that the results I have obtained in these cases during the last few years since I have adopted Mr. Allingham's method of dealing with these abscesses have been much more satisfactory. By this method of treatment, even in cases where I have been able to demonstrate a rectal communication at the time of the operation, I have secured healing without the formation of a permanent fistula. Therefore, in any case of inflammation of the tissues of the ischio-rectal fossa, whether the evidence of abscess be clearly demonstrated or not, I follow the method which is recommended by Mr. Allingham, which consists in etherizing the patient and placing him in the lithotomy position, after having located the position of the indurated tissue or abscess; and a rectal examination by means of the finger will often assist in locating the position of the abscess. A free incision several inches in length is made through the tissue, outside of and parallel with the fibres of the external sphincter muscle, and the incision is gradually deepened until the pus cavity is reached. It is then slit up to the length of the skin incision and the cavity is explored with the finger, breaking down any loculi which tend to divide up the abscess cavity, and so make one cavity of the abscess. The cavity of the abscess is next washed out with a 1:2000 bichloride of mercury or 1:60 carbolic acid solution, and is then packed with strips of iodoform gauze and a pad of the same gauze is placed over the wound and over this a pad of bichloride of mercury cotton is laid, and the dressing is secured in position by a T-bandage. An opium suppository is introduced into the rectum and the bowels are kept quiet for three or four days.

The dressing is not removed for two or three days, and at this time the packing is usually loose and can be removed without difficulty, and after its removal the cavity is injected with peroxide of hydrogen, and it is then irrigated with a 1:2000 bichloride of mercury solution, and next the cavity is lightly packed with strips of iodoform gauze and the wound is covered with a pad of iodoform gauze and bichloride cotton. The same steps are observed at subsequent dressings, which are made at intervals of two or three days, and the cavity usually heals rapidly by granulation and contraction, and in a few weeks it is usually completely healed.—*Dr. H. R. Wharton, in The College and Clinical Record.*

### BISMUTH FOR BURNS.

Von Bardeleben recommends the topical employment of bismuth in burns, except those of a slight character, in which weak solutions of silver nitrate and elastic collodion suffice. He thinks severe burns requiring early amputation may also be excepted from burns amenable to the bismuth treatment.

After thoroughly cleansing the burned sites, the author washes them with three per cent. carbolic, or three per cent. acid salicylic solution. He then removes any blisters and their contents that may be present, employing antiseptic precautions while so doing. Then he thoroughly powders the whole region with: ℞. Bismuth subnit., subtilliss., pulv. amyl, *aa.*, ʒxiiss.; M. f. pulv. This he dresses with layers of cotton, which, when saturated by the secretions, are removed, except the lower-most layer, which is left to maintain exclusion of the air. This dressing may be left *in situ* one or two weeks, even a month, thus avoiding all pain incident to change of dressing. In most cases the pain of the original injury disappears within a few hours after applying the bismuth.

In burns of the face von Bardeleben uses no other dressing. He disinfects the burn, removes the bullæ, and powders the wound with bismuth. The crusts developed are gradually removed in a week or two by grease, especially where they are most adherent; that is, at hairy surfaces, such as the eyebrows, beard, etc. If any denuded surfaces remain, they soon are covered with skin under the employment of silver nitrate.—*Exchange.*

---

### THE PRESENT POSITION OF GALL-BLADDER SURGERY.

Czerny after a general consideration of gall-bladder surgery, presents the following conclusions:

1. Gall-stones require operation, if they cause frequently repeated or lasting trouble.

2. Empyema of the gall-bladder imperatively demands operation, as does hydrops, if it gives serious trouble.

3. The typical operation for gall-stones consist in incision, removal of the stones, and suture of the gall-bladder; in this, however, the abdominal wound should be drained for a short time.

4. If the cystic duct is closed, if the gall-bladder is the seat of inflammation, or the contents are greatly altered, then a temporary gall-bladder fistula must be made.

5 Extirpation of the gall-bladder is indicated only in cases of severe inflammatory or carcinomatous involvement.

6. When the ductus choledochus is closed, the operation is absolutely indicated if the strength of the patient will permit. If one does not succeed in removing the stone or obstruction, then it is recommended to produce a fistula between the gall-bladder and duodenum.

7. The best incision for gall-bladder operations is an ȷ-shaped cut; the vertical limb lies in the linea alba, and the horizontal part runs towards the right just below the level of the umbilicus.

8. The danger to life in gall-stone operations will be probably less than in operations for vesical calculus.—*Epitome of Medicine.*

---

### STRANGULATED HERNIA—WHEN TO OPERATE.

In former years there was some question as to just when it was justifiable to operate—that is in other words, just how far you were to allow your patient to go towards "death's door" before giving him relief. I regret to say that there are still a fair number of physicians who do not feel any alarm for their patient until fecal vomiting begins, and believe that it is then quite time enough to talk of surgical measures. They fondly imagine that they are acting in the patient's interest by trying everything else before the knife. Then, again, there are a large number too timid to operate themselves and not conveniently situated to call a surgeon. To these two causes many lives are sacrificed every year.

I look upon delay as by far the most dangerous feature of the case. In thirty-one cases of strangulated hernia in private practice I have lost three cases, and I am sure that two of those might easily have been saved by an early operation. This good record is due to two causes: First, that I have seen the cases early; and, second, that I have never left my patient until the hernia was reduced.—*DeGarmo, Internat. Med. Mag.*

---

### FOR SHOCK FOLLOWING ABDOMINAL OPERATIONS.

For cases of slight shock following abdominal operations, Dr. Edward P. Davis, of Philadelphia, recommended the following as a stimulant:

℞. Elixir ammonii valerianat., f℥ j.

Spirit frumenti, f℥ j.

Aquæ bullient, f℥ ij.

M. Give by rectal injection every two hours.

—*Lancet-Clinic.*

